# Supplementary figures and images for: Increasing plant group productivity through latent genetic variation for cooperation
Source: PLoS Biol. 2022 Nov 29;20(11):e3001842. doi: 10.1371/journal.pbio.3001842 (PMC9707777; doi:10.1371/journal.pbio.3001842)

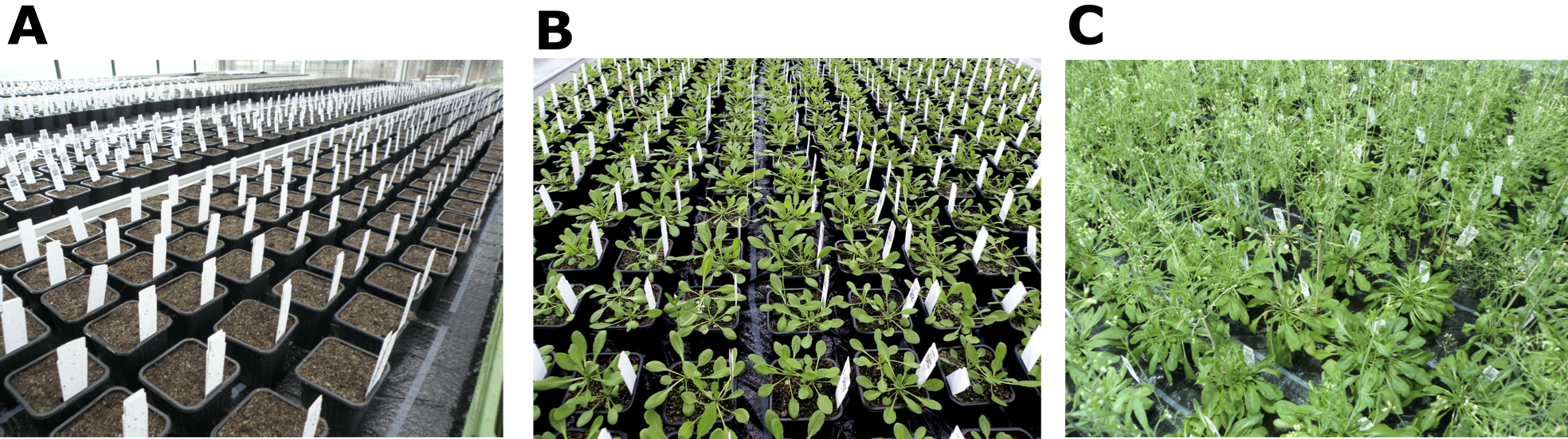

Supplement: S1 Fig — (A-C) Photos show the experiment at sowing (A), midway through the experiment (B), and at harvest day (C). (TIFF) [file pbio.3001842.s001.tiff]

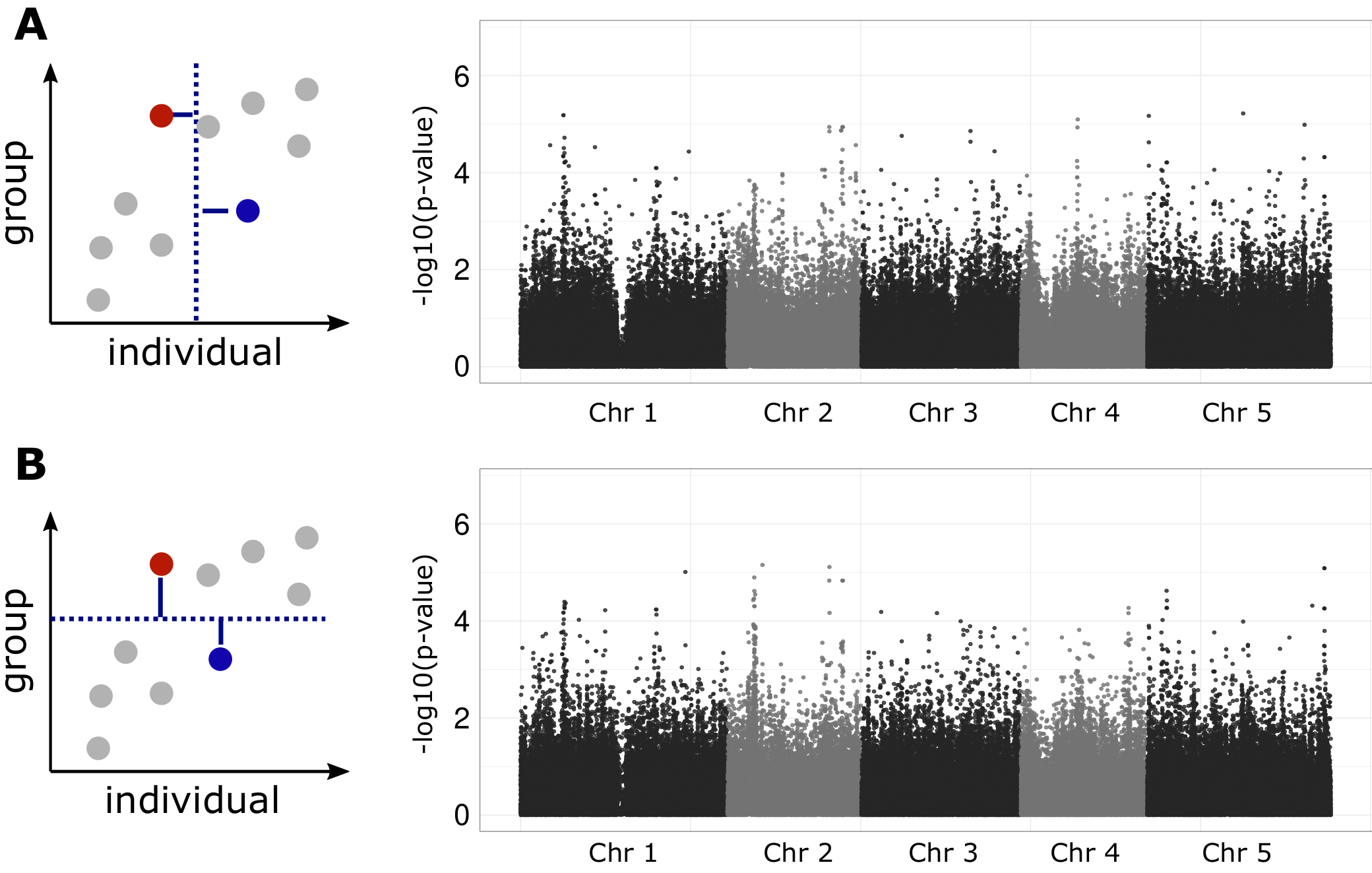

Supplement: S2 Fig — Association tests for variation in (A) average individual performance across mixtures or (B) average monoculture performance. Data available at https://zenodo.org/record/6983283, file competition.csv. (TIFF) [file pbio.3001842.s002.tiff]

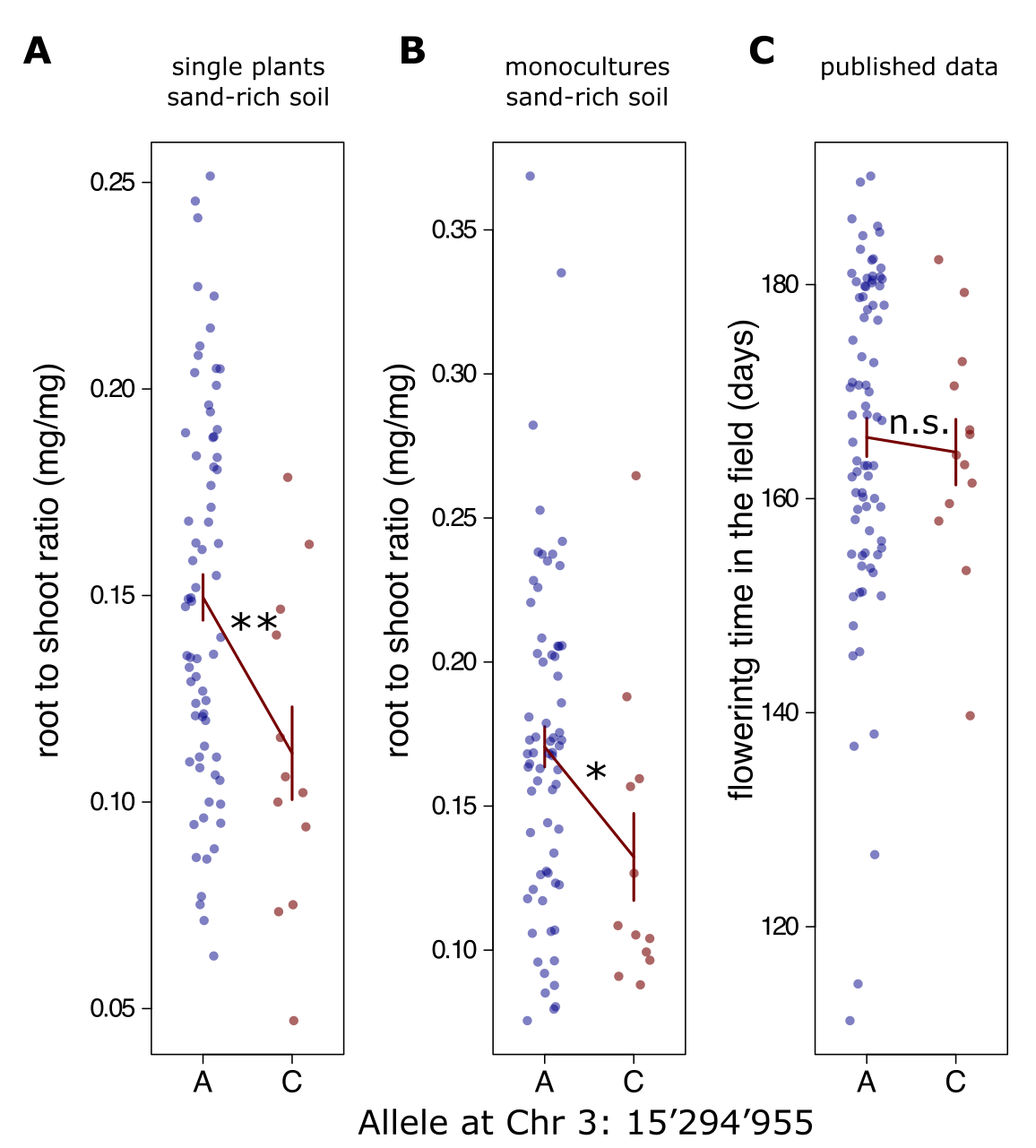

Supplement: S3 Fig — (A and B) Shoot-to-root ratios for genotypes grown in monocultures (A) or as individual plants (B) in an independent experiment and on sand-rich soil are shown. (C) Published data of genotypic means in flowering time in the field [36]⁠. Bars show mean ± SEM. ** = ANOVA p < 0.01; * = ANOVA p < 0.05; n.s., not significant. Data available at https://zenodo.org/record/6983283,file sand.csv, and at https://arapheno.1001genomes.org/phenotype/86/. (TIFF) [file pbio.3001842.s003.tiff]

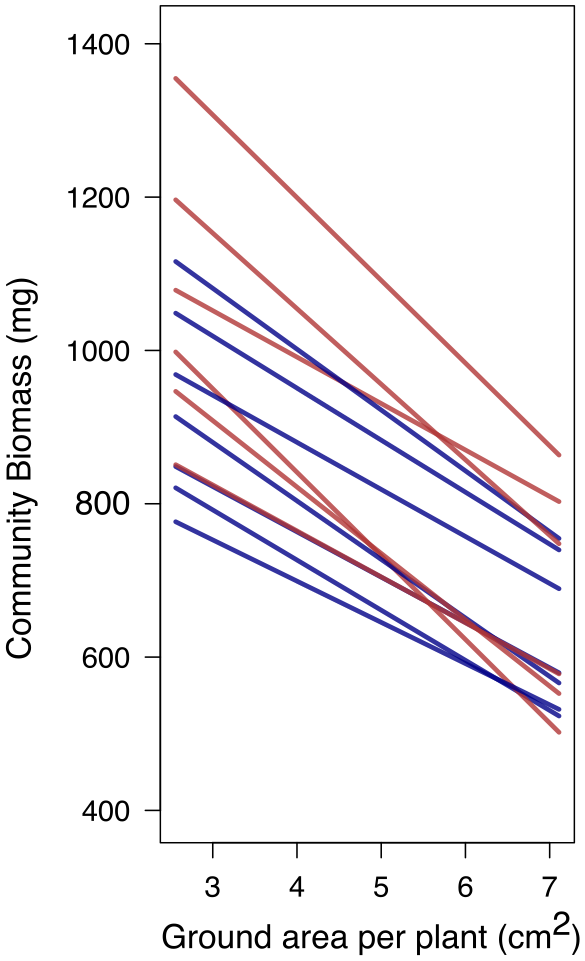

Supplement: S4 Fig — Lines represent reaction norms or genotypes carrying different alleles at Chr 3 SNP 15’294’955; red lines: cooperator allele carriers; blue lines: competitor allele carriers. Data available at https://zenodo.org/record/6983283, file densitygrad.csv. (TIFF) [file pbio.3001842.s004.tiff]

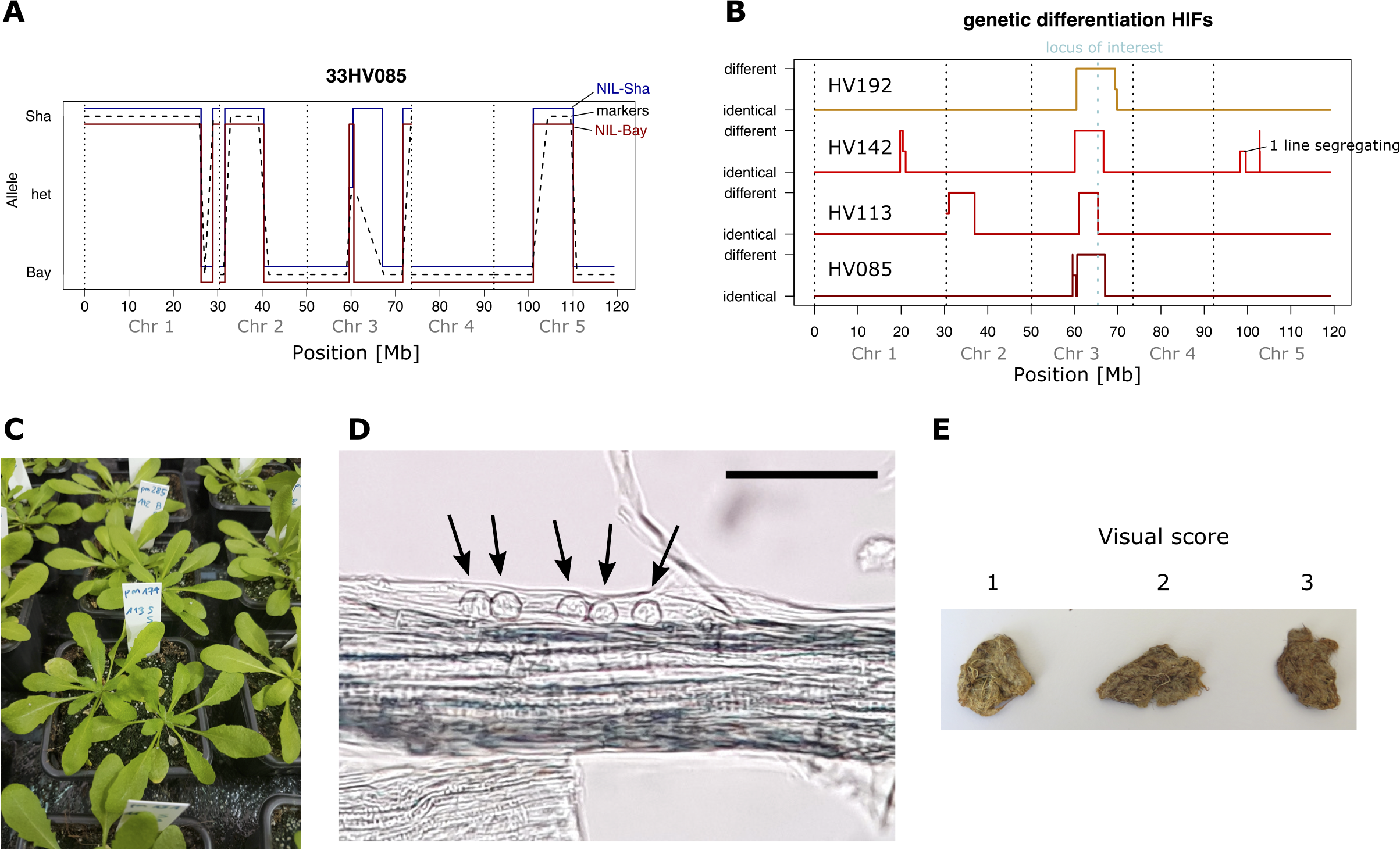

Supplement: S5 Fig — (A) Genome reconstructions of two near-isogenic lines (NILs) from the 33RV085 parental RIL. Viterbi paths of each NIL are shown in red (Bay-allele at Chr3:15’294’955) and blue (Sha-allele), together with published marker data of the parent (dashed line). (B) Genetic differences identified in whole-genome comparisons between the pairs of near-isogenic lines. All NIL pairs differ from each other at Chr3:15’294’955 (light blue: “locus of interest”). (C) Earliest sign of shoot-level disease expression in line 33RV113 carrying the Sha-allele. (D) Microscopic image of dried root samples containing resting spores of the obligate parasite Olpidium brassicae (arrows). Scale bar = 50 μm. (E) Visual scoring scheme of root browning for washed and dried root samples from the competition experiment. Scoring was performed fully blinded to the sample ID or genotype. (TIFF) [file pbio.3001842.s005.tiff]
