# Supplementary material for: Increasing plant group productivity through latent genetic variation for cooperation
Source: PLoS Biol. 2022 Nov 29;20(11):e3001842. doi: 10.1371/journal.pbio.3001842 (PMC9707777; doi:10.1371/journal.pbio.3001842)
Supplement: Attachment — Submitted filename: Response to reviewers_final.pdf [file pbio.3001842.s007.pdf]

Dear Dr Roberts,

We thank you and all reviewers for the thorough reviews and suggestions for improvements. We also thank you for the opportunity to submit a revised manuscript, which we do here. The main changes we made are:

- we modified the figures to improve contrast and readability. This should make them more suitable for visually impaired readers.

- we clarified the definition of cooperation and added references to kin selection and associated phenomena.

- we added further clarifications about possible interpretations of the genetic mapping (e.g. why we could not detect significant associations with monoculture performance) and about the limitations of our data to infer specific mechanisms.

We followed-up on the concerns raised about the robustness of our analyses. Specifically, we removed potential data outliers and repeated all related analyses. These analyses differed only very little from the original analyses. We therefore propose to leave the data presentations and analyses unchanged.

A point by point reply to the specific points raised by the reviewers is provided below. Our responses are all highlighted in black, and line numbers refer to the revised manuscript.

We think that our manuscript has benefited substantially from this revision and hope that it now meets the standard for publication.

Yours sincerely

Samuel Wuest, for all authors

**Reviewer #1 (Remarks to the Author):**

*This exciting manuscript provides a mechanism for screening multiple genotypes for cooperation, does so using a set of mapping lines, and finds an association between a putative cooperative allele and lowered root allocation and increased disease resistance. The question is excellent and important.*
*This manuscript justifies and illustrates an approach to finding 'cooperative' alleles for crop breeding, in which 'cooperative' alleles are defined as alleles that increase stand fitness at the cost of individual fitness. They then screened for 'cooperative' alleles by growing plants in same accession vs. different accession pairs, and looked for the accessions that fit that fitness pattern. They were able to map the alleles and demonstrate that they are associated with root allocation and disease resistance. There is somewhat of a mismatch in the main experiment, though, between the goals of the researchers and what their methodology ends up measuring. I am seeing one experimental weakness and some conceptual issues.*

*Their definition of cooperation from game theory (line 38) meets the definition of altruism in evolutionary theory. 'Cooperative' alleles are defined as alleles that increase stand fitness at the cost of individual fitness. This definition is actually a definition of altruism, or costly help. In reference 22, I used altruism to refer to costly help, and cooperation to refer to within-species help that benefits the helper. Cooperation can be defined more broadly as any kind of within species helping, costly or not. I strongly argue that authors should reference and discuss their definition of cooperation, given the confusion in the literature.*

Thank you for pointing out this potential source of confusion. We fully agree that cooperation is a complex phenomenon that can be approached from different angles, including evolutionary theory.

In our work, we adopt a pragmatic, operational perspective, and focus on a game-theoretical notion and measure cooperation as difference in pay-off between genotype monocultures and the same genotype under heterogenotypic competition. This definition is formally linked to the payoff matrix describing the prisoner's dilemma (as shown in Figure 6), and complies with the classification scheme given in reference 22, because "costs" of helping for the helper only arise in the context of selfish neighbors, but in the context of cooperative neighbors brings reciprocal benefits. To improve clarity, we have modified the respective statement (line 38), which now reads "Our method is based on the game-theoretical premise that alleles increasing cooperation benefit the monoculture group but are disadvantageous to the individual when facing non-cooperative neighbors"); this also fits the definition of Axelrod and Hamilton (Ref 28 in the revised manuscript).

We have further edited the following paragraph (revised ms, lines 94-100), which now reads: "Cooperative strategies in natural plant communities may be selected for under certain circumstances (e.g., spatial clustering of closely related individuals) [23,24], but in situations where competing individuals are unrelated, the evolutionary stable strategy generally is one that maximizes individual performance (and resource use) to the detriment of the group as a whole [25–28]. However, if genetic variation for cooperation exists in nature, for whatever reason, it could potentially be leveraged in breeding [29].".

We have also added additional references to the literature on kin selection and kin recognition, as described below.

*I did not argue in my review [22] that "plant cooperation among unrelated individuals rarely evolves in natural systems because plants often grow in communities of unrelated individuals, a*

*setting under which cooperative phenotypes have low fitness" (lines 96-98). Plants also often grow in groups of related individuals because most dispersal is local - so-called viscous populations (Hamilton). Facilitation, helping between species at the same trophic level, is frequently found in nature.*

Our statement did not reflect reference 22 properly, and we have edited it accordingly, as described above (re-written paragraph lines 94-100).

We agree that local dispersal can generated clusters of closely-related individuals, and that this can promote kin selection. However, we think that there are also many examples of the contrary effect (e.g. height in trees as in Ref 25, root overproliferation in close proximity to neighbors as in Ref 46), and that some selection regimes used in breeding tend to promote non-cooperative traits.

More generally, our results provide only limited insight into processes in natural systems. Our main focus is on the question whether cooperative traits can be found in plants (regardless of the reason they occur), and whether these can be exploited in human-driven selection, e.g. in crop breeding.

*Moreover, the authors may have wanted to find altruism towards unrelated individuals, but their experimental design is the same one used in kin recognition studies. Plants were grown in same- and different-accession pairs in the experimental design. The goal was to create a monoculture of equally cooperative or competitive phenotypes, but there is a considerable body of evidence on plant identity recognition that needs to be considered. Many researchers have measured the fitness consequences of growing with relatives (File et al. 2012), though to my knowledge not at the fine scale seen in the manuscript. This manuscript should reference (Milla et al. 2009) who measured the fitness consequences of growth in pairs of kin and strangers in Arabidopsis.*

We agree that kin recognition is a mechanism that can promote cooperation in monogenotypic stands. We now mention this possibility explicitly (see below) and refer to the suggested literature, which indeed is very interesting.

Our take on cooperation, however, is more pragmatic, and we deliberately focus on the pay-off matrix in Prisoner's dilemma-like settings to quantify cooperation and not on particular mechanisms. The method we propose is deliberately agnostic to the underlying mechanisms.

Our results provide only limited insight into processes in natural systems, but we fully agree that the evolutionary processes that lead to cooperation in nature are important and interesting, they just are not the focus of study.

Also, note that we did not quantify specific cooperative responses (e.g reduced allocation to competitive structures) towards clones (congenotypic neighbors, "kin") and towards more distantly-related non-clones (heterogenotypic neighbors). Kin recognition would imply different degrees of cooperation in these two settings, which may have occurred, but it may as well be that the cooperative genotypes we identified were equally cooperative under heterogenotypic competition and that this was the reason they performed poorly in these mixed communities.

However, because the topic of kin recognition is closely related and interesting, and readers will be familiar with it, we refer to this possibility:

Line 429-432: "Another idea is to incorporate kin selection schemes into breeding, which could also select for crops that recognize relatives [22,24,55]. Kin recognition could promote cooperation in monocultures, but it is currently unclear how widespread kin recognition is in plants [57]. "

Here, we make reference to the suggested publications (including Milla et al 2009), but also refer to a newly published perspective paper on the topic and its relevance in breeding (Biernaskie JM. Kin selection theory and the design of cooperative crops. Evol Appl. 2022;00: 1–10), for a more in-depth discussion.

*The results in this manuscript are not necessarily a consequence of kin discrimination. Altruism can evolve in viscous populations and as an outcome of multilevel selection (Weinig et al. 2007). Direct benefit helping (=cooperation as defined in [22]), which is 'within-species facilitation' is understudied but is known to occur. The methods in the present manuscript would potentially find these types of helping.*

We agree, and have included these references and modified definitions, as discussed above.

*The authors used a factorial design to create the accession pairs, with all possible combinations of the 97 panel accessions with 10 tester accessions and itself. The analysis is not an anova; instead the authors use performance in the same accession pair as the dependent and average performance with the tester accessions as a predictor. However, the consequence is that performance in mixed stands is better replicated (20 pots per accession) than performance in monoculture (2 pots per accession) so that the standard error is higher for estimates of performance in monoculture. I'd class this as a weakness rather than a fatal flaw since it doesn't violate the assumptions of regression. Plant growth is variable, and more replication of the monoculture would be better in future studies.*

We agree with this point. On the other hand, a higher replication of the mixtures was required for non-statistical (biological) reasons because we aimed to represent the range of competitive strategies present in the accession panel – the goal was to identify particularly cooperative genotypes relative to the population mean.

The higher replications of mixtures is the main reasons we regressed monoculture performance (with the larger statistical error) against mixture performance (with a smaller statistical error). We could also have run a major axis regression, but it would have made little difference. Also, note that this regression merely serves to derive a metric of cooperation, which then is analysed in separate statistical models.

*Detailed comments follow:*

*1. I like the introduction with the clear discussion of ideotype breeding. It motivates the study well.*

Thank you for the positive feedback.

*2. Line 130-134. Reference for cooperation in game theory?*

We have added an explicit reference to the paper by Axelrod and Hamilton, and also cite Weiner et al (Ref 30) here.

*3. Line 137, figure 1. What is the justification - theory and references - behind the estimates of the G-I tradeoff? This important approach needs more discussion.*

The G-I tradeoff has been originally described by Peter Jennings and colleagues as part of their work to breed the Green Revolution rice variety IR5 (“miracle rice”) at IRRI. The idea was picked up again and extended and formalized in a more recent paper by Jacob Weiner and colleagues (Ref 3). We believe it is best to refer to these sources for an extended discussion and theory behind, and provide again the link to the game-theoretical treatment of the costs and benefits of cooperation depending on social context. We have clarified this as follows:

Lines 124-127: “The core principle of our method is to measure the performance of a set of genotypes in different “social contexts” [30], allowing for the quantification and subsequent genetic mapping of variation in a G-I trade-off (Weiner [30] discusses this trade-off in detail).”

*4. In figure 1, the axis indicates group performance in monoculture, while line 572 suggests the model was fit using "Monoculture biomass per individual (i.e. total average monoculture biomass divided by two). I note that in Figure 1, average individual performance in mixtures is mostly slightly less than half that of a pair of same-accession plants. Cooperative accessions are evaluated as above average rather than as having higher performance in monoculture than mixture. That one point with low performance in mixture and over 3X higher performance in monoculture may be very influential. Here the value of more replication in monocultures is clear.*

We agree and therefore made sure that our analyses are insensitive to the inclusion of individual points. First, the GWAS using the 1001 whole genome sequencing data did *not* include this point, because this genotype was not part of the 1001 panel (i.e. this analysis is based on only 68 genotypes). Figure 2B shows that the results are very comparable. Second, we state that “the SNP with the strongest association resides in the center of a transposon-rich region and explained approximately 25% of the variation in the genotypic G-I trade-off values". We tested the effect of removing said point, and found that this resulted in a reduction of variation explained to 21%. This demonstrates that the results are robust and do not depend much on individual data points.

*5. Use of synonymous terms - accession and genotype. Use one consistently.*

We replaced the term “acccession” with the term “genotype” throughout the manuscript

*6. In reading the experimental design, I found that it was unclear by how it is written that each panel genotype is paired with each tester genotype, and had to do some math to confirm that it is a factorial design.*

We clarified this sentence (line 145: “We further grew individuals of all genotypes in full factorial combination with an individual of each of ten tester genotypes (genotype mixtures; **Fig. 1A,B**; Supplementary **Fig**. **S1**).*”)*

We also clarified this in the methods-section (Lines 512-515):

“Competition experiment: pairs of individual plants were grown in small pots (6×6×5.5 cm) in a factorial design in which the 97 genotypes of the panel were each grown together with one of ten tester genotypes (Bay-0, C24, Col-0, Cvi-0, Ler-1, Sav-0, Sf-2, Shahdara, St-0, Uk-1).”

*7. The figures are not colourblind friendly or printer friendly, since often they only indicated point identity by red and blue. Ideally they should use shape and colour to distinguish groups, with colourblind friendly colours*

Thank you for pointing this out. We made the following changes

- Figure 1 C: we changed the symbols of the minor allele (red) to open circles, so that they can be distinguished without referring to colors

- Figure 3A: we additionally labeled the two lines to make them easier to distinguish

- Figure 3B: we now use different line types for the two allele groups, and added a legend

*8. Line 230. Why was area per individual used rather than plants per square meter, when the legend indicates performance across a realized planting density gradient? Plants per square meter is more typical in ecology and can be readily compared to other studies. The increased performance with density indicates maximum yield per pot was not realized, which is good.*

Both metrics hold the same information content (one is the inverse of the other). However, using density resulted in a non-linear relationship and a less favorable residual distribution. We now write: Lines 603-606: *“*For the density gradient experiment, productivity was modeled in dependence of the fixed terms area per individual, allele, plus their interaction (we chose area per individual

(available space) instead of density (the inverse of available space) because the relationship with density was non-linear).*"*

*9. Line 261+ "We confirmed that this pattern of lower root-to-shoot ratio in cooperative genotypes holds across environments by conducting an independent experiment, where we explored this effect in both monocultures and in isolated individual plants, and in a different soil type (Fig. 4C, and Supplementary Fig. S3). Overall, our findings indicate that reduced relative root growth is part of a genetically fixed strategy associated with enhanced cooperation." Root allocation tends to be plastic in Arabidopsis to nutrients. The lateral root number increases with strange exudates compared to kin and self exudates (Biedrzycki et al. 2010) .*

We agree that root allocation generally is a plastic trait; however, our results suggest that the identified allelic variation also influences trait means. We found reduced root allocation in different contexts (monocultures and single plants, on different soil types). We could not measure root allocation in mixtures (because it was not possible to separete root biomass of the competing genotypes), and we therefore could not analyze the role of plasticity in cooperative interactions. Nevertheless, our results support the finding of reduced relative root allocation in the system of interest – the monogenotypic group.

To improve clarity, we now reviews the text and include the indicated reference as follows (Lines 271-273): "Overall, our findings indicate that relative root growth is associated with the identified genetic variation, and that reduced root allocation may be part of a strategy associated with enhanced cooperation [37].*"*

*10. Line 295, line 461. This conclusion that any variation in cooperation must be latent seems very weak to me. It ignores considerable theory and some evidence that cooperation and altruism can be favored in plants.*

Thank you for bringing this point to our attention. We agree that there are circumstances in which cooperation can be selected for in plants, in natural settings. To acknowledge this fact, we have amended the text (as discussed above) and added additional the suggested references, including a very recent perspective paper on the matter "Biernaskie JM. Kin selection theory and the design of cooperative crops. Evol Appl. 2022;00: 1–10."

We do not claim that *any* variation in cooperation must be latent. However, in the specific case it could well be. We simply argue that cooperative traits may arise as a "by-product" of other genetic or evolutionary processes.

*11. The resistance evidence is post-hoc. It is intriguing but not a planned study. It is also difficult to follow. Some of that is presentation. Fig 6 does not clearly indicate the reward structure is for the focal plant (rows) as determined by the strategy of both the focal plant (rows) and the neighbour plant (columns). The Wikipedia explanation of the prisoners dilemma payoffs is more clear than what is presented here. The text vs. background contrast is very low for the darkest background and I cannot read the numbers. Some of that is that is that the design is complex and needs more attention from the reader. I recommend it be published as a separate paper.*

The finding that a disease can modify the competitive outcomes of between allele carriers is, of course, completely serendipitous (especially since the detected parasite must have invaded the experiment without planning). However, we think that serendipity has an important place in scientific discovery. In particular, we think we were extremely "lucky" to make an observation that provides a plausible explanation for the maintenance of natural variation in the population. We therefore believe that these findings are well worth presenting.

We are aware that the experimental design and its outcome are complex. We explicitly included a pointer to the classical prisoner's dilemma in the text (317-320): "Further evidence for the idea that the allele for cooperation is advantageous in environments with pathogens emerged from an

additional experiment in which we quantified benefits of individual alleles (payoffs) in well-defined competitive interactions modeled according to the classic prisoner's dilemma."

and changed text colors in Fig. 6 to improve readability.

*12. Lines 511 - 518.The authors should be clear that the stands are single accession.*
We now write (new ms lines 525-527): "Density gradient: to test for decreased self-inhibition in plants carrying the cooperation-associated allele, genotype monocultures with different individual densities (3×3, 4×4 or 5×5 plants on 8×8cm area) were constructed."

*13. The analysis section does not include root allocation. I do promote the methods that use an additive formulation (Coleman et al. 1994; Gedroc et al. 1996; McConnaughay and Coleman 1999).*

We have added this information to the methods section as follows (Lines 597-602):

"To test for the dependency of the G-I trade-off value on relative root allocation, we linearly regressed the trade-off value against root-to-shoot ratio and tested for significance of the slope term. We did not transform the axes because the relationship was sufficiently linear. Note that analysis on a log-log scale, as frequently used in allometric analyses, was not appropriate here because the G-I trade-off value was centered around zero (i.e., not systematically positive). "

*14.Reference 51 is the same as 21*
Thank you – corrected !


*Biedrzycki, M., Jilany, T., Dudley, S. and Bais, H. 2010. Root exudates mediate kin recognition in plants. Communicative and Integrative Biology, 3: 1-8.*
*Coleman, J.S., McConnaughay, K.D.M. and Ackerly, D.D. 1994. Interpreting phenotypic variation in plants. Trends in Ecology and Evolution, 9: 187-191.*
*File, A.L., Murphy, G.P. and Dudley, S.A. 2012. Fitness consequences of plants growing with siblings: reconciling kin selection, niche partitioning and competitive ability. Proceedings of the Royal Society B-Biological Sciences, 279: 209-218.* http://www.doi.org/10.1098/rspb.2011.1995.
*Gedroc, J.J., McConnaughay, K.D.M. and Coleman, J.S. 1996. Plasticity in root/shoot partitioning: optimal, ontogenetic, or both? Functional Ecology, 10: 44-50.*
*McConnaughay, K.D.M. and Coleman, J.S. 1999. Biomass allocation in plants: ontogeny or optimality? A test along three resource gradients. Ecology, 80: 2581-2583.*
*Milla, R., Forero, D.M., Escudero, A. and Iriondo, J.M. 2009. Growing with siblings: a common ground for cooperation or for fiercer competition among plants? Proceedings of the Royal Society of London, Series B: Biological Sciences, 276: 2531-2540.*
*Weinig, C., Johnston, J.A., Willis, C.G. and Maloof, J.N. 2007. Antagonistic multilevel selection on size and architecture in variable density settings. Evolution, 61: 58-67.* http://www.doi.org/10.1111/j.1558-5646.2007.00005.x.


**Reviewer 2**

*World food production relies on our ability to increase crop yield. Identifying genes underlying increased crop yield is an extraordinarily important research area. The authors present the results of an interesting study demonstrating a method to identify genotypes within populations that are more productive (cooperative) when gown together in high density stands. I believe this is an important study that could have wide ranging impacts on crop production. I have some comments*

*on the current manuscript, most of which are relatively minor.*

*1) The main thing that I would have recommended be done differently is to present results for reproductive output or fecundity. Most crops are seeds or fruits, and reproductive output may have been a better measure than shoot biomass of performance under competition. It is likely that reproductive output is correlated with shoot mass, and the results would probably be quite similar. However, competition can induce plants to increase their allocation to reproduction at the cost of reduced future vegetative growth.*

We agree that for many crops, it would be more relevant to study reproductive output (though we have now also include a pointer to crops where biomass is a relevant trait, e.g. forage grasses, see below). Our main goal was to develop a method that allows quantifying cooperative traits and discovering related alleles. With Arabidopsis, focusing on seed yield would have been difficult. We also think that reproductive output will be correlated with shoot mass. To make it clear that the target trait is a matter of choice, and that metrics other than shoot mass could have been chosen, we write:

Lines 101-105 “Here, we present a methodological framework for the discovery of such alleles. We aimed for such a framework to be as general and unbiased as possible, in order to detect **yield or biomass gains** (or any other performance measure) that emerge from any type of cooperation, including for unknown resources and through unidentified traits.”.

Lines 141-143 “Aboveground dry matter production (shoot biomass) served as the measure of performance (Materials and Methods), **but any other target characteristic such as agricultural yield could also be used**.”

Lines 470-472: “In this work, we focused – primarily **for the sake of simplicity** – on a **model population of *Arabidopsis thaliana***, and **on above-ground biomass (which is a target trait for some crops, such as forage grasses)**.”

*The authors build a compelling case that the alleles associated with cooperation in Arabidopsis are also associated with pathogen resistance, and this can explain the maintenance of cooperation alleles in the population. If competition induced more efficient reproduction, and this was an important adaptive response in plants, then it may be common for alleles associated high reproductive yield under competition (since these alleles wouldn't have been strongly selected against as many cooperation alleles would have been). This might be worth mentioning in the manuscript.*

This is an interesting argument, which may apply in some scenarios. However, since we did neither study seed production nor the particular mechanism underlying it, we prefer not to include such speculations. It also is somewhat against the experience from breeding that shows that in most grain crops competition reduces group-level seed output per unit ground area.

*2) Lines 85-86 - We don't yet have a great understanding of the traits associated with competitive ability in plants. These traits may be associated with poor competitive ability, but they may not be.*

We fully agree with this point. Here we argue that ideotype breeding has severe limitations exactly for this reason. We edited this sentence and now write “**Traits desirable for the breeder** are, for example, a short stature, erect leaves, and a compact root system [12,13,19].”.

*3) Figure 2b - this relationship seems to be driven by the two points with the highest root-shoot ratio. Consider transforming, or demonstrating that the relationship is significant without these two points.*

Two points indeed stand out. However, they do not drive the relationship. We have repeated all analyses (i.e. group comparisons in Figure 4A, right panel; relationship in Figure 4B) after removing these points, and found that the results differ very little. Essentially, group A (Fig 4A, right) is so large that these two data points aren't influential.

Also, note that while these two points could in principle increase the effect in panel A, they would have the opposite effect to reduce significances in panels B and C (they tend to make the regression line flatter).

*4) Line 326 - I may have missed this in the methods, but how was the reduction in competition measured?*

This explanation was ambiguously formulated. We have changed it to read (lines 332-335):

"However, such payoffs are difficult to quantify directly, and therefore we refer to payoffs as the reduction in above-ground biomass of plants competing relative to plants grown individually (e.g., **a small reduction in biomass under competition** = high payoff)"

*5) Line 382 - increasing density tends to decrease growth. This allele tends to allow plants have high growth relative to the genotypes with the more competitive allele.*

Exactly. The group benefits of cooperative alleles increased with density.

*6) Line 498 - provide details of pot size.*

We have added this information as requested.

**Reviewer 3:**

*Wuest et al. present a set of elegant experiments to map loci underlying individual-group tradeoffs in Arabidopsis, and propose these approaches as a novel framework to identify loci for breeding. I really enjoyed reading this paper -- clearly thought-out experiments, good presentation, and well written. I also like the authors' overall argument about the importance of loci underlying cooperation, and understand the choice of Arabidopsis in terms of feasibility. Overall I'm very positive about this work!*

Thank you!

*I do have a few of clarifications and questions. Likely some are from my own misunderstanding or misreading, so I apologize ahead of time for things I may have missed.*

*First, I am not sure I agree with the authors that their general framework is feasible for most crops. For a breeder evaluating progeny from some cross, doing this full set of experiments would be very time consuming and costly and is not easy to set up in a field scenario. Greenhouse work costs more and less scaleable than field trials. I really have trouble seeing how this experimental approach could approach industry-scale breeding.*

This method indeed is labor-intensive and unlikely to be regularly applied in the process of selection in a breeding population.

One key application we see is pre-breeding. Our method could be used to identify alleles promoting cooperation, and these alleles could then be introduced into the breeding population. Once these alleles are identified, they could support marker-assisted breeding methods and biotechnology.

One could argue that – when selecting for yield in high-density stands – alleles promoting cooperation should also be selected for. However, not all selection occurs in high-density stands,

and in cases where the relevant genetic variation is not present in the breeding population, selection for yield will not pick up cooperative traits.

*Second, I think I could use some more clarity on the value of this approach over just selecting for group yield/fitness alone, or looking for loci by mapping fitness in a bunch of monogenotypic trials (which is e.g. what is done in maize as the authors cite in the discussion).*

*I was surprised to see their results that suggest the chr3 locus is not found in GWAS for overall group fitness, and would like some more explanation on what they think is going on. It seems to me that, under a simple additive model, given a sufficiently powered data set you should see the effect of this locus even if it's sometimes found in low-vigor genotypes. So the argument that "cooperator" alleles can't be found in low-vigour genotypes doesn't seem to hold for me -- you can find "tall" alleles even though they are sometimes found in short individuals, for example. Instead, perhaps it is something along the lines of the nonlinearity between individual/group fitness or genotype/phenotype the authors bring up in the discussion (line 440-442) what's driving this disconnect between the overall group fitness and ability to map this locus? If so, is some kind of nonlinearity to be expected more commonly for "cooperator" loci than for other traits? Some additional explanation/guidance/speculation here would be helpful, since at least my initial reaction was that mapping/selecting for group yield is already in fact what lots of breeding is doing, making me wonder why this approach was necessary. The authors empirically show that you get a different result, but it would be helpful I think to dig in a bit more as to why and how often we might expect this to be the case.*

These indeed are important points, and we have thought about these a lot ourselves. The reason no association of monoculture performance (or individual performance in mixture) is found with the identified locus on chromosome 3 is that the effects of these alleles are masked by large variation in general vigour among genotypes. This likely is the case because growth is a complex polygenic trait that results from many small effects and a wide range of underlying physiological processes. However, these polygenic effects, whatever their nature, seem to affect the performance of a genotype both in monoculture and in mixture in similar ways, i.e. this variation appears as variation along our heuristic and not as variation along the orthogonal axis that reflects the G-I-tradeoff.

We made this point clearer by amending the text as follows (lines 201-207):

"Direct mapping of untransformed data, i.e., of variation in either individual or monoculture group biomass alone, did not reveal any significant association with this locus (Supplementary **Fig. S2A,B**). This was expected, because such an analysis fails to separate general vigor from the G-I trade-off value that we used here to quantify cooperative properties. We did not find any other associations, likely because monoculture productivity is a complex polygenic trait and depends on a broad range of underlying processes."

We also agree that a genomic approach (e.g. GWAS) could in principle pick up the relevant variation in monoculture performance. However, this would require much larger data sets (= more statistical power) to isolate the effect of the cooperator allele from all the other variation in growth – a problem that our approach solves by "re-directing" these different sources of variation onto separate orthogonal axes.

On the other hand, genetic variation in cooperation can indeed also be leveraged in genomic selection. We actually already state this in our manuscript (lines 432-437): "Finally, genomic predictions are increasingly adopted to predict a genotype's merit purely based on its genome,

whereby prediction models are trained on yield variation of high-density stands of pure varieties. These approaches have arguably contributed to historical and contemporary yield advances, and implicitly or explicitly include elements that selected to some extent for cooperative traits [22].".

However, our method is simply more specifically tailored towards social traits, by adjusting for general effects of size and thus reducing "residual" variation in GWAS analyses. This has several advantages, including the possibility to identify genes that underlie social traits. Once identified, these could ultimately be very rewarding for biotech.

*Finally, is it really fair to call the chr3 locus a "cooperator" allele given the disease results where it leads to increased competitive fitness too (lines 349-352)? Is this a cooperation locus or just a locus that affects disease resistance and has a pleiotropic effect decreasing root/shoot ratio (or does it just affect root size alone)?*

Our framework is one for the discovery of alleles underlying cooperation, which is why we refer to it as "cooperator" allele (cooperation is, in this application, the trait of interest). We agree that pleiotropic effects are involved, and therefore write already in the abstract (line 43) "The allele likely affects a pleiotropic gene, since we find that it is also associated with reduced root competition but higher resistance against disease.".

More generally, we agree that it remains unclear how exactly (e.g. in physiological terms) cooperation is promoted. As mentioned in the replies to reviewer 1, we also acknowledge that the effects via altering root-to-shoot ratio are somewhat speculative, but at least they agree with what has been published for other types of cooperation.

*Minor Points:*

*The extended LD around the locus on chr3 sure looks like this could be a structural variant. I recommend the authors look up the region in assembly comparisons of thaliana lines to see (I looked in* [https://academic.oup.com/mbe/article/38/4/1498/6008718#235101396](https://academic.oup.com/mbe/article/38/4/1498/6008718#235101396) *but doesn't look to me like there's an inversion of sufficient size there, though I didn't check their insertion/deletion results).*

This is an interesting question, and it would certainly be relevant if we were to clone the specific genes and formulate more specific evolutionary hypotheses. For the present manuscript, we feel that right now we do not have sufficient information that would justify going into the specific genetic details of this variation.

We see our work as a proof-of-concept study to demonstrate the usefulness of our framework to discover genetic variation underlying cooperation, and not so much as a investigation of the specific cooperator allele that we have found in this Arabidosis panel. Based on our focus, the next step would be to apply this method to agricultural crops.

*Does root-shoot ratio by itself predict cooperation or group fitness?*

Yes, variation in root-to-shoot ratio predicts variation in the G-I-trade-off, as shown in Figures 4B und C. Please also refer to the replies given to reviewer 2 regarding the robustness of this result.
