# Supplementary material for: Increasing plant group productivity through latent genetic variation for cooperation
Source: PLoS Biol. 2022 Nov 29;20(11):e3001842. doi: 10.1371/journal.pbio.3001842 (PMC9707777; doi:10.1371/journal.pbio.3001842)
Supplement: Attachment — Submitted filename: Revision.pdf [file pbio.3001842.s008.pdf]

Dear Dr. Roberts,

Thank you very much for the helpful review and the opportunity to submit a further revision of our manuscript.

To addressed the remaining issues, we have implemented the following main changes:

- We have further edited the sections where we refer to the possibility of kin recognition and kin selection to improve clarity, and have added further references.

- We have edited the Figure 1 (legend and axis labels) to improve clarity

- We have added the links to data sources of the figures

More generally, we find it important to highlight that the method for the discovery of cooperative genotypes that we present here is deliberately agnostic to the reason why these traits exist in the first place. This is so 'by design' and we believe that this is a particular strength of our approach. Our study was not designed to and provides only very limited opportunities to analyze mechanisms of kin recognition. However, we agree that kin recognition is a related topic and may play a role in some scenarios. We therefore refer to this possibility in the text, thanks to the reviewer's input (incl. 5 references to the reviewer's own work). However, this really has the character of a side topic, for the reasons outlined above, and we would like to keep it that way. Otherwise, we would create the false impression that our findings (and the applicability of our method) is contingent on kin recognition as underlying mechanism promoting cooperation.

We hope that with these changes and explanations the manuscript now meets the standards for publication in PLoS Biology.

Yours sincerely,

Samuel Wuest

(on behalf of all authors)

Requests (in blue):

a) Please attend to the remaining requests from reviewer #1.

This is an important and interesting paper. It reads very well. I strongly suggest the Figure 1 be better documented - see comments below. I also have a few detailed comments that I think would improve the manuscript. The introduction and methods could better integrate the finding from multiple researchers that plants demonstrate plasticity to the relatedness of neighbours (kin recognition). The authors are screening for cooperative alleles expressed in monoculture. Because crops are grown in monoculture, this is entirely appropriate. However, they should acknowledge that there is a potential for a kin recognition response affecting performance between monocultures and mixed pots. Though they don't analyze the monoculture/mixed pot curve, the markedly higher fitness for accessions grown in monoculture vs. mixed pots is predicted by kin selection theory, and contributes to kin recognition research, and so itself interesting. That they found a tractable single locus cooperation allele as well demonstrates the strength of their approach. While looking for kin recognition is arguably irrelevant for crops grown in monoculture, kin recognition should not be ignored for this study.

We are grateful for these inputs and the kin selection and kin recognition literature indicated. We have further expanded the respective sections and think that our manuscript now is very clear in this respect. See below for specific details.

More generally, we find it important to stress that the framework we present here for the discovery of cooperative genotypes and the alleles underlying cooperation is agnostic to the reason why these traits exist in the first place. This is intentionally so ('by design') and we think that this is a very important strength of our approach. Our study was not designed to analyse mechanisms of kin recognition, and it also is not our aim to delve into the mechanisms underlying the evolution of kin recognition. We, however, agree that it is important to point out this possibility, and we have implemented this in the revised text as suggested.

1. Line 93 -97. Altruism (costly help) should only evolve in related groups, but cooperation broad sense evolves because it will benefit the cooperating individual. See (Lehmann and Keller 2006) (Dudley 2015). Be more exact in the use of terminology.

We have revised our statement to include this case. We now write:

> *"Cooperative strategies in natural plant communities may be selected for under certain circumstances (e.g., spatial clustering of closely related individuals, or in cases where relatives recognize each other through kin recognition) [23,24], but in situations where competing individuals are unrelated, typically the evolutionary stable strategy is one that maximizes individual performance (and resource use) to the detriment of the group as a whole [25–28]. "*

We have further carefully checked our use of terminology, and think that it is consistent with the literature related to plants and in particular crops. Because it is important to clearly define the meaning of cooperation, we use and refer to the game-theoretic framework introduced in the seminal work by Axelrod and Hamilton (1981). In the text and Figure 5, we use the concept of the payoff-matrix to describe cooperative strategies and their benefits to the individual in different social contexts. In the frame of this concept,

cooperation has a cost when facing non-cooperative opponents relative to the situation facing cooperative individuals.

Our terminology is consistent with the use in the papers by Prof. Dudley, who for example writes "*we treat* ***cooperation as the inverse of competitiveness****, in that plants may cooperate by competing less, or act selfishly by competing more.*" (Dudley, Murphy & File, Funct. Ecol. 2013). Along these lines, we for example write "*any excessive investment into competition in the presence of neighbors undermines what is, from an agronomic perspective, an efficient use of resources at the group level. In plants,* ***cooperative growth strategies*** *thus* ***may be characterized by a relative insensitivity to the presence of neighbors****, or* ***even a reduction in the development of competitive structures****".*

As explained above, we conducted greenhouse experiments with the primary aim to identify alleles that underpin cooperative traits. We did not study their evolution in a natural setting and therefore think that it is not warranted to speculate too much about this topic – although this clearly is an intriguing question!
In our experiment, we observed the association of disease resistance with the more cooperative strategy. Based on our experimental data, the most parsimonous explanation for the existence of this allele is that the associated disease resistance provides a (direct) fitness benefit to the carriers. It may or may not be that kin recognition also plays a role for the maintenance of this allele in nature – testing this would require additional studies and data about the population structure, which we do not have.

2. Line 98. Population structuring is common in plants as most dispersal is local even with mechanism for long distance dispersal. Plants commonly self and/or clone, resulting in high relatedness. Therefore, conditions that select for altruism may happen frequently, depending on the species ecology.

We agree with this, and the importance of this is indeed probably underestimated in the evolutionary literature. In our work, we developed and tested a framework to discover alleles that underpin cooperative traits; our goal was not to study the evolution of altruism and cooperation and we therefore only state briefly that we (and as we believe many others) do not necessarily expect cooperative traits to be all that common in plants.

3. Line 129-137 makes arguments for the experimental procedure entirely on the basis of functional traits and game theory, ignoring kin selection theory and the potential for plasticity to the relatedness of neighbours. Because same accession is the proxy for same functional traits, kin recognition and constitutive cooperation are confounded. Because the goal is to identify cooperation in monoculture, this is not a problem. However, not discussing these issues will result in readers aware of the work to question the methodology.

Indeed, we deliberately chose a procedure rooted in game theory that is agnostic to the specific mechanisms that underly cooperation. In our view, this is a strength, not a weakness, of the method. Kin recognition effects – if they occur – do not in any way change the validity of our findings.

As explained, it is important to us that our method works independent of the specific mechanisms. However, to connect to readers familiar with kin selection theory, we refer to it in several places (e.g. lines 94-99; 421-424, 443-449; references 22, 23, 24, 37, 54, 56, 58, 59). On the other hand, we think that is would be misleading to expand this discussion

further, first, because this might create the false impression that cooperation (and our findings) are contingent on kin recognition mechanisms (which they aren't), and second, because our data really does not allow their analysis.

4. The legends for figure 1 does not make it clear whether the authors are plotting mass of average single plant in monoculture or average pair of plants in monoculture vs average of a single plant in mixture. The legend needs to explain what the 'group' is. Lines 580-582 indicates that the analysis used the average individual single plant in monoculture. If the authors are plotting the average individual single plant in Fig 1, then monoculture plants are substantially larger than mixed culture plants. I would like to know if they found any size differences between mixed and monoculture plants.

This was indeed not very clear, and we have now modified the legend to address this point. We also have changed the axes labels, which now read ("Monoculture group performance (mg per two individuals)" and "Mean individual performance across mixtures (mg per individual)".

We deliberately chose to plot the group value for the monoculture to highlight that this is a property of a collective of individuals of the same genotype. For the statistical analyses, we expressed all data per individual. However, this does not matter for the results at all given that this is a simple scaling factor of 0.5.

With respect to average size differences among monoculture and mixture individuals: The tester genotypes are not a truly random sample of the whole population with respect to size. The role of the testers was to represent a range of competitive strategies, but also because they had been crossed as parents in various mapping populations (convenient for follow-up analyses) - and we seem to have chosen rather vigorous genotypes (on average) for this role. This means that genotype's individual performances in mixtures should be slightly downward biased, simply because there is a slight sampling bias in the tester lines used. We have now made this point explicit in the methods-section, as follows:

Lines 531-535: "These tester genotypes were chosen because they constitute the parents of different publicly available recombinant inbred line populations, i.e., a genetic resource that allows for certain genetic experiments (for example, isolating near-isogenic lines from heterogeneous inbred families, see Figure 6)."

To analyse this data with respect to kin recognition (which here is not our focus), one would have to consider the genetic relatedness of the genotypes grown together, which we did not do (and again, which was not our focus). However, the data will be (if published) openly available for such analyses.

5. Line 425-426. I actually disagree with using kin selection in crop breeding itself, since crops are grown in single cultivar groups, and don't experience competition with non-relatives of the same species, so there is little opportunity for kin selection or advantage to kin recognition. However, studies of kin recognition in wild plants are another way of picking up potential cooperative traits, e.g. (Dudley and File 2007) also identifies root allocation as a competitive/cooperative trait.

We have changed the wording of the corresponding paragraph. It now reads:

> *"Another idea is to incorporate kin selection schemes into the breeding process (e.g., in the early cycles of breeding programs where individual genotypes*

compete with co-segregants), which could also select for crops that exhibit cooperative traits [24,56,58]. Though it is currently unclear how widespread kin recognition is in plants [59], kin recognition studies in wild populations are another avenue that could promote the identification of cooperative traits in plants [22].”

The added reference 58 by Montazeaud et al. Proceedings Biol Sci. (2020) shows the merit of kin selection schemes for improving a target trait (reduced height) that increases yield. A recent perspective on kin selection schemes for breeding and implications (Ref 56, Biernaskie, Evol. Applic. 2022) on the topic has been added during the previous revision.

b) Please provide a blurb, according to the instructions in the submission form.

Blurb: “This paper proposed a simple method to identify plant genotypes that express more cooperative versus more competitive traits based on how they grow in different social environments, and applies this method to a genetic study with *Arabidopsis thaliana*.“

c) Please address my Data Policy requests below; specifically, we need you to supply the numerical values underlying Figs 1C, 2ABC, 3AB, 4ABC, 5ABC, 6D, S2AB, S3ABC, S4, S5AB, either as a supplementary data file or as a permanent DOI’d deposition like Zenodo, Dryad, Figshare, etc.

We have added the required information: the dataset generated as part of this study is publicly available at https://zenodo.org/record/6983283, and includes a sample script that demonstrates how to calculate and interpret the G-I-tradeoff from our data.

d) Please cite the location of the data clearly in all relevant main and supplementary Figure legends, e.g. “The data underlying this Figure can be found in https://zenodo.org/record/XXXXX”

We have added the corresponding infos to the figure legends.
